# A European inventory of common electronic health record data elements for clinical trial feasibility

**DOI:** 10.1186/1745-6215-15-18

**Published:** 2014-01-10

**Authors:** Justin Doods, Florence Botteri, Martin Dugas, Fleur Fritz

**Affiliations:** 1 Institute of Medical Informatics, University Münster, Albert-Schweitzer-Campus 1/A11, D-48149 Münster, Germany; 2 Integrated Information Sciences, Development, Novartis Pharma AG, CH-4002 Basel, Switzerland

**Keywords:** Electronic health record, Data elements, Feasibility criteria, Clinical information system, Clinical trials

## Abstract

**Background:**

Clinical studies are a necessity for new medications and therapies. Many studies, however, struggle to meet their recruitment numbers in time or have problems in meeting them at all. With increasing numbers of electronic health records (EHRs) in hospitals, huge databanks emerge that could be utilized to support research. The Innovative Medicine Initiative (IMI) funded project ‘Electronic Health Records for Clinical Research’ (EHR4CR) created a standardized and homogenous inventory of data elements to support research by utilizing EHRs. Our aim was to develop a *Data Inventory* that contains elements required for site feasibility analysis.

**Methods:**

The Data Inventory was created in an iterative, consensus driven approach, by a group of up to 30 people consisting of pharmaceutical experts and informatics specialists. An initial list was subsequently expanded by data elements of simplified eligibility criteria from clinical trial protocols. Each element was manually reviewed by pharmaceutical experts and standard definitions were identified and added. To verify their availability, data exports of the source systems at eleven university hospitals throughout Europe were conducted and evaluated.

**Results:**

The Data Inventory consists of 75 data elements that, on the one hand are frequently used in clinical studies, and on the other hand are available in European EHR systems. Rankings of data elements were created from the results of the data exports. In addition a sub-list was created with 21 data elements that were separated from the Data Inventory because of their low usage in routine documentation.

**Conclusion:**

The data elements in the Data Inventory were identified with the knowledge of domain experts from pharmaceutical companies. Currently, not all information that is frequently used in site feasibility is documented in routine patient care.

## Background

Many clinical studies have difficulties in recruiting the required number of patients within the specified time frame and subsequent protocol amendments are costly. Between a third and half of the studies meet their recruitment numbers and over half take longer than planned to reach their goal [1,2].

At the same time, clinical documentation is increasingly carried out in electronic health record (EHR) systems and, therefore, huge amounts of data are stored in an electronic format. EHRs are used for routine patient care but can also be used to support the identification of eligible patients for clinical research [3].

If recruitment could be optimized by a better selection of clinical research centers, and if better trial protocols could be created through an improved and more accurate site feasibility analysis, clinical studies could be completed faster and be more cost-efficient. It is common practice during feasibility analysis that pharmaceutical companies ask physicians how many patients they treat under certain conditions in a certain period of time. The physicians then estimate the number of suitable patients per site. However, how these results are compiled is non-transparent for the study sponsor. An improvement of the site feasibility analysis could be achieved through re-use of EHR data to generate more reliable patient count estimates for pharmaceutical sponsors of clinical studies and sponsors of investigator initiated trials (IITs) alike. If the protocol designers know how the feasibility numbers come about, they can redefine their criteria to improve the protocols.

Even though EHRs are being adopted in more and more hospitals, data is not necessarily reusable. Data can be captured fully structured, semi-structured or in free text. Structured data are documented during routine patient care through the use of national value sets or terminologies, but it is currently unclear what kind of data, besides data for reimbursement, are available across European EHR systems. It is also unclear how much of these data are relevant and specific enough for clinical research and which data elements are most relevant for feasibility analyses of clinical studies.

To tackle those issues, the IMI [4] funded project ‘Electronic Health Records for Clinical Research’ (EHR4CR) [5] aims to support clinical trials, including site feasibility analysis, through the re-use of EHR data. The project runs over four years (2011 to 2014) and, being a public-private partnership, consists of 33 partners from industry and academia. Clinical partners are located in France, Germany, Poland, Switzerland and the United Kingdom. Scenarios which will be addressed are ‘clinical protocol feasibility’, ‘patient identification and recruitment’, ‘clinical trial execution’ and ‘adverse event reporting’. EHR4CR is focused on the following disease areas: oncology, inflammatory diseases, neuroscience, diabetes, cardiovascular and respiratory diseases. The project will utilize existing or specifically created clinical data warehouses and connect those databases to the ‘EHR4CR Platform’ in secure technical ways and comply with European data protection laws.

As part of the workpackage ‘Pilots’ (WP7), we aim to obtain an overview of the data content and frequency in EHRs which allow electronic support for protocol feasibility. Our objective is to develop an inventory of available core data elements of European EHRs for all the disease areas of the EHR4CR project. These data elements have to be relevant for clinical research according to clinical trial experts from the European Federation of Pharmaceutical Industries and Associations (EFPIA). Our motivation is to foster secondary use of EHR data for research and our research question therefore is: What are the common data elements in Europe relevant for site feasibility analyses and what is currently available in EHRs to create a valid and EFPIA accepted inventory?

## Methods

### Data element

The term ‘data element’ is used in several contexts with multiple possible meanings. The ISO/IEC 11179 Standard defines a data element in Part 1 [6] as follows:

‘A data element is produced when a representation is associated with a data element concept. The representation describes the form of the data, including a value domain, datatype, representation class (optionally), and, if necessary, a unit of measure.’

In the following, we focus on a consented definition with a data element concept comprising two parts. The first part is assigned to identify groups of related data elements (data group), for example ‘Findings’, while the second specifies the datatype in more detail (data item), for example, ‘Weight’. Links to Unified Medical Language System (UMLS) [7] codes are provided to identify the underlying medical concepts. Representations, as defined in ISO/IEC 11179, with value domains, data types and units of measurement for each data element were not specified for the Data Inventory, because data sources with different languages were analyzed. Instead, examples of typical values were provided. Table 1 shows an example of such a data element.

**Table 1:** Data element example

**Data element concept**	**Example**	**Consensus definition**	**Link**
Findings/Weight	80 kg	The weight of a subject.	http://ncim.nci.nih.gov/ncimbrowser/pages/concept_details.jsf?dictionary=NCI%20MetaThesaurus&code=C0005910

Example for the definition of data elements. Data element concepts consist of a data group and data item part. The elements also contain an example, a definition and a link to the NCI Metathesaurus referencing its UMLS concept.

### Data inventory

The Data Inventory is a catalog of data elements. Every data element consists of a data group and data item part, which together correspond to ISO 11179’s data element concept. Elements also contain a sequential ID, an example for a possible data value, a definition and a link to the UMLS code of its medical concept.

### Material

The Data Inventory was created from an initial list provided by the pharmaceutical companies with data elements they consider most important for their studies. In addition, the inventory contains data elements from 17 studies from acute or chronic diseases in oncology, neurology, diabetes, cardiovascular and inflammatory diseases (see Table 2). These studies were selected from the EFPIA companies in the EHR4CR project and had finished their feasibility phase as of end 2011. The selection excluded Phase I and non-interventional studies. Additional criteria for the selection were that the studies should have run at least at one EHR4CR data provider site (the participating hospitals are: Assistance Publique - Hôpitaux de Paris, Friedrich-Alexander-Universität Erlangen-Nürnberg, Hôpitaux Universitaires de Genève, Kings College London, Medical University of Warsaw, Université de Rennes, University College London, University of Dundee, University of Glasgow, University of Manchester, Westfälische Wilhelms-Universität Münster) - preference was given to those studies that ran at the most - and that each EFPIA company (participating companies are: AMGEN, AstraZeneca, Bayer Health Care, F. Hoffmann-La Roche Ltd, GlaxoSmithKline, Johnson & Johnson, Lilly, MERCK KGaA, Novartis Pharma AG, Sanofi-Aventis) was represented with at least one study. With the exception of one company, the criteria could be met for the current version of the Data Inventory.

**Table 2:** Overview of the companies, the numbers and disease areas of studies used

	**Cardiovascular**	**Diabetes**	**Inflammatory**	**Oncology**	**Neurology**
AMGEN				1	
AstraZeneca				1	
Bayer Health Care	2				
GlaxoSmithKline				3	
Johnson & Johnson		1		1	
MERCK KGaA					2
Novartis Pharma AG	1		1		1
F. Hoffmann-La Roche Ltd.	1				
Sanofi-Aventis				2	

Data sources used for the project depend on the access to the systems by the local partners. In total, 15 EHRs were surveyed, because some sites used data from their whole EHR and others data from one or more departmental subsystems, for example specific systems for breast cancer or diabetes.

### Methods

**Figure 1:**
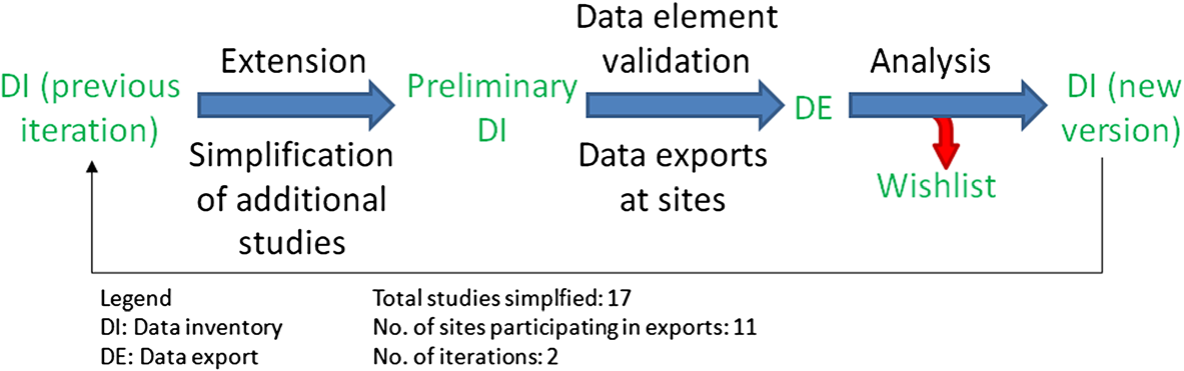
**Main steps of the iterative approach to create the data inventory.** A list of data elements (DI (previous iteration)) is extended by data elements of simplified eligibility criteria. The availability of data elements from the extended list (Preliminary DI) then get validated through data exports (DE) at the sites, which afterwards get analyzed. Data elements which are hardly used or not available at the sites get removed from the DI and are added to the wish list. The remaining elements form a new version of the Data Inventory (DI (new version)).

The process to create the Data Inventory was iterative and consensus driven. An overview of the main steps of our iterative approach is summarized in Figure 1. Face-to-face meetings and telephone conferences were carried out to achieve common understandings and agreements. Between ten and 30 people attended the meetings and calls, depending on their availability. As a starting ground, pharmaceutical companies were asked to provide a list of the most commonly used data elements for the feasibility phase, based on their own personal experience. Elements were grouped by their context to create the data groups and afterwards the initial list was iteratively extended by data elements from a total of 17 studies. The data elements from the study protocols were extracted by expert-driven, manual ‘simplification’ of eligibility criteria [8]: feasibility and recruitment experts from the companies removed unnecessary text phrases or unimportant information until the core information for ‘feasibility criteria’ remained. ‘Patient with confirmed deep vein thrombosis’ was, for example, simplified to ‘Diagnosis/Text: deep vein thrombosis’. Data exports at the eleven EHR4CR sites were conducted to capture the availability of each element (available yes/no) and the frequency of documentation (measured in relative percentages) at the source systems. To distinguish between data elements that are not available in EHRs and those that are simply not documented - both could in theory be represented as ‘0%’ - availability and frequency of a data element were captured separately. To avoid privacy concerns and allow for comparability between sites the relative percentage of each element was captured instead of absolute numbers. Relative percentages were calculated by first identifying how many patients had an entry in the EHR for each data element and then dividing it by the number of all patients seen in the respective time frame. These exports were then analyzed by creating rankings and heat maps, displaying the general availability and usage of the elements by using different colors. Microsoft Excel [9] was used for the analysis and creation of the heat maps. The heat maps were created using the conditional formatting feature.

For each iteration, consensuses on the data elements in the Data Inventory were agreed, for example, splitting the element ‘Child bearing status’ up into four separate elements (‘Currently pregnant’, ‘Pregnancy number’, ‘Menopausal status’, ‘Lactation’). Other decisions were that elements were moved to a separate list, referred to as ‘wish list’, because they are not available or hardly used at any of the sites.

Each of the data elements was manually reviewed by a peer group of ten pharmaceutical and informatics experts. The background of the pharmaceutical experts is in feasibility assessment/management, drug safety, data management/analytics and clinical operations. The review was needed to determine whether the elements were viable for the feasibility stage and whether the meaning of the element name was clear to all peer group members. Once a common understanding was reached, definitions were identified and added to each data element.

## Results

In the following section, the Data Inventory in its current version is described as well as the wish list and result from the latest data exports.

### Data groups

The data groups define the context of the data elements. Data groups which a data element can belong to are:

demographics, medical history, diagnosis, procedure, findings, laboratory findings, medication, scores and classifications, or patient characteristics.

### Data Inventory

The Data Inventory in its current version is composed of 75 elements. It consists of 5 demographics, 4 diagnosis, 7 findings, 41 laboratory findings, 8 medical history, 7 medication and 3 procedure data elements. The definition of each element contains a link to the corresponding UMLS Concept Unique Identifiers [10] at the NCI Metathesaurus (NCIm) [11] and a textual description. In case the NCIm reference did not contain a textual definition, a suitable one was created by the expert group.

**Figure 2:**
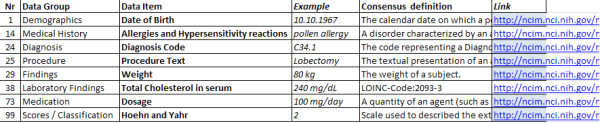
**Examples from the data inventory.** Each element in the data inventory contains a sequential number, the data group and item, an example of a possible value, the textual definition and the corresponding NCIm link.

An overview of examples from the Data Inventory, containing data elements from each data group, can be seen in Figure 2. The whole Data Inventory can be found in the additional material (Additional file 1).

### Wish list

Data elements that showed through the data exports to be not available or hardly documented were removed from the Data Inventory and put on a separate list which contains 21 data elements. The rarely available data elements in EHRs are listed in Table 3.

**Table 3:** Data elements of the wish list

**Data group**	**Data items**
Findings	QTc interval, Left ventricular ejection fraction
Laboratory findings	MAGE-A3 status
Medical history	Trial title, Inclusion date, End of participation date, Current method of contraception, Vaccines, HIV status, Lactation
Patient characteristics	Willingness to participate in clinical trials
Scores/classifications	Date of score/Classifications, Karnofsky-score, Eastern Cooperative Oncology Group -performance status, TNM-classification, New York Heart Association - status, Response Evaluation Criteria in Solid Tumors, Hoehn and Yahr, scale, GRID-Hamilton Depression Rating Scale, Mini-Mental State Examination, Unified Parkinson’s Disease Rating Scale Section 1

The wish list contains data elements that are currently not, or very rarely available, in European EHRs, but that are frequently requested in study protocols.

### Availability of data elements

Data exports captured the availability and the usage of the data elements. Elements which are highly available are from the data groups demographics, diagnosis, procedures and the majority of the laboratory findings. Rarely documented are the elements from the groups ‘scores and classifications’ and medical history, with the exception of allergies and hypersensitivity reactions. Medication and findings data elements are generally available, but not in all of the systems.

**Figure 3:**
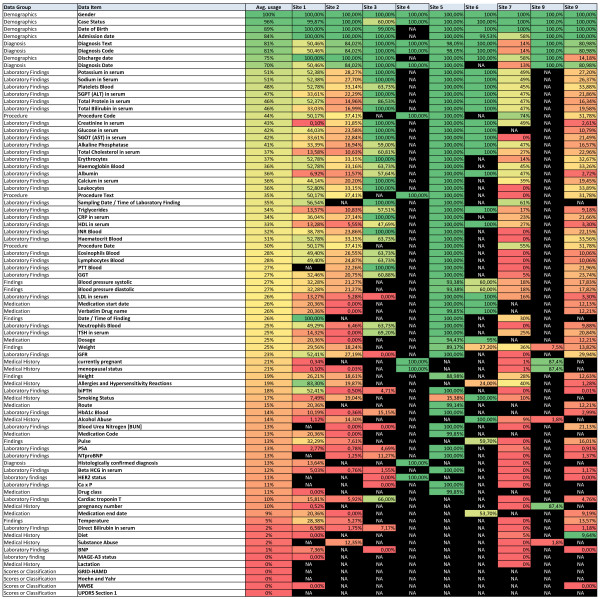
**Heat map of the data exports from the data inventory current version.** The first two columns describe the ISO 11179 data element concept (data group/data item). The third column shows the average usage of the data element over all sites while the following columns (site 1 to site 9) display the frequency at the individual sites. The Data Inventory is ordered by the average usage sorted in descending order from most available to least. The frequency ranges from 100% (dark green) to 0% (dark red). Data elements that are not available at a site are shown as Not Available (NA) (black).

The color-coded heat map (Figure 3) gives an overview of the general availability of the data elements of the Data Inventory. The six least available data elements in this figure were moved to the wish list after the analysis of the heat map.

## Discussion

The overall goal of this work was to identify data elements that are needed for site feasibility analysis in clinical studies and are at the same time commonly documented in European EHR systems. The heat map was created to determine the availability across the data provider sites. The coloring of each cell was considered a good means to give an overview of how frequently each element is documented and especially, highlight those which are generally not used. Widely available data elements are from data groups demographics, diagnosis, procedures and laboratory findings. Under-documented elements are those captured in the wish list which are from the groups ‘scores and classifications’ and medical history. We chose to use own groups instead of using Weng *et al*./Luo *et al*.’s [12,13] semantic classes, because our focus was not only on clinical trials, but also on EHRs. With the data groups, we also wanted to indicate where data could be found in EHRs. For example, procedures are used in Europe for Diagnosis Related Groups (DRG). Consequently, diagnostic and therapeutic procedures would be covered by the same data group. Each element was reviewed by feasibility and recruitment experts and contains a definition and link to the NCIm to ensure a clear understanding and avoid confusion of the exact meaning. Value lists, for example, diagnoses that are frequently used for the project’s disease areas, are not specified and out of scope of this work, therefore they were not taken into consideration. The focus is on availability and frequency of data elements at EHR4CR pilot sites, so only examples for values are given. The high availability of the elements from the aforementioned data groups is most likely because they are needed for reimbursement and quality management. Laboratories have started structuring their data very early, because in most cases special laboratory information systems are connected to the main EHR. Because the data have to be available there, laboratory findings are also highly available. The exact reasons, however, were not investigated. Despite the fact that a lot of laboratory findings are available in EHRs, many laboratories do not yet use standard terminologies like Logical Observation Identifiers Names and Codes (LOINC). Classifications like the International Statistical Classification of Diseases and Related Health Problems 10th revision (ICD10) on the other hand, are standard in European EHRs. That is also the reason why we used general data elements for diagnoses and procedures, because it is generally easier to identify diagnosis and procedures data in EHRs than it is to find non standardized data elements.

Data elements were ranked according to the availability of the exports, so that elements of low availability could be identified. The wish list contains those additional data elements that are relevant for clinical research, but not documented in a structured manner during routine care. To enhance secondary use of data for clinical research, EHR systems could be extended to allow a structured documentation of these elements like the Eastern Cooperative Oncology Group (ECOG). This would not necessarily result in more documentation work, but rather in a different representation of the same content. Instead of free text ‘patient is bedridden’ an ECOG score of 4 could be documented. We assume that scores like the Mini-Mental State Examination (MMSE) and medical history elements like ‘current method of contraception’ are primarily documented for research purposes. There is generally no direct incentive for documentation of intensive scales with many data elements, especially considering that physicians already spend equal or more time on patient documentation than on direct patient care [14,15]. This might indicate why those elements dropped out of the Data Inventory and into the wish list. Likewise, this might be the case for the medical history data elements ‘Trial title’, ‘Inclusion date’ and ‘End of participation date’ which are also related to research. We further assume that data elements from the data group medical history are more frequently documented than our exports show, but most likely in free text and not as structured data. Natural language processing is not within the scope of EHR4CR and therefore only structured data were used. The number of data elements that can be found in free text was not further investigated and is subject for future research.

To create a valid and sponsor (EFPIA or IIT)-accepted Data Inventory, we decided to compile the list through an iterative and consensus driven process with partners from academia and strong participation from domain experts of European pharmaceutical companies.

The EFPIA partners in the project are among the largest researching pharmaceutical companies in Europe, with many studies each year. This way both sides added their perspectives and increased the acceptance of such a list. The international character of the focus group and the validation at several university hospitals makes the data inventory meaningful beyond national borders. No references on settings of average European hospitals were found so whether the data exports at non-university hospitals would have resulted in similar availability numbers cannot be stated. An iterative approach was chosen as a pragmatic way to see if our method was feasible and to improve single steps of the process as we went along. One example is, that we verified the availability of data elements in the first data export in percentage groups (6: 100%, 5: < 100 to 75%, 4: < 75 to 50%, 3: < 50 to 25%, 2: < 25 to 10%, 1: < 10 to > 0%, 0: 0%, N/A: not available), while in the second round, exact relative percentages (for example, 98%) were requested. Each iteration included the simplification and identification of data elements from eligibility criteria of study protocols. This was done by feasibility and recruitment specialists from the pharmaceutical companies themselves. Analysis and processing of eligibility criteria has been done by other groups [12,16,17], but our aim was not to follow a specific representation or create a new format. We wanted to extract the most important information out of those free text criteria and display them in a simple, comprehensible way for all stakeholders. By doing so, we were able to identify the underlying data elements and add new ones to the Data Inventory.

The subsequent validation exports at eleven data provider sites with varying disease specific focuses were either done on whole EHRs or on subsystems of the hospitals, depending on available data sources. This is also the reason why some sites have many black cells in Figure 3, when they used a specialized departmental subsystem instead of the whole EHR. A bias by those sites that used specific subsystems can therefore not be excluded, but while general elements like ‘date of birth’ were not negatively affected, disease or gender specific elements were influenced positively. ‘Medical history/menopausal status’ was, for example, seldom documented in the majority of the systems, but was always available in a breast cancer system.

Because the Data Inventory contains data elements with clear definitions, it can be used as a reference for important data elements when new forms are created in EHRs. Through both lists, the Data Inventory and the wish list, it is clearer what to expect if EHRs are to be used for clinical research. In general, EHR systems could be accredited for their compliance with catalogs of important data elements in the future. This could demonstrate that the respective product is more suited to support secondary use of health care data for clinical research than non-compliant EHR systems.

The simplification of eligibility criteria was a manual task focused on clinical trial feasibility. It is possible that different criteria would have been identified by other people. The Data Inventory is created as part of the EHR4CR project and therefore, the studies were selected to include each company and each data provider site. This means that other studies, other disease areas and different companies might also have resulted in different data elements. However, given the large number of involved countries, hospitals and trial experts, this Data Inventory represents an important consensus. Given that EHR4CR covers six major disease areas we assume that the Data Inventory will in general cover a large part of clinical studies.

### Related work

In the following we compare the Data Inventory against work that relate to ours.

Weintraub *et al*. [18] compiled a list of 100 cardiovascular ‘data fields’ that were identified from existing data standards as being a ‘base set of terms with maximal value’ to specified criteria. The list is intended to be used in EHRs and facilitate secondary use, but was not validated with data exports from EHR systems.

A comparison of the data fields and the data elements of the Data Inventory showed that because of the different scope and definitions of data elements, both lists cannot readily be compared. Out of the 100 data fields, 20 exactly match data elements of the Data Inventory, while 46 are not directly captured in the Data Inventory, but would rather be values of our data elements and 34 do not match at all. An example of a data field that would be a value in the Data Inventory is ‘diabetes’ which we would consider a value of the data element ‘Diagnosis/text’. Table 4 shows in more detail how the data fields correspond to the data elements.

**Table 4:** **Comparison of the Data Inventory with US cardiovascular data fields**[18]

**Number of data fields matching data elements of the Data Inventory**	**Exact match**	**Data field as value of a data element**	**No match**
History and physical examination elements	8	24	5
Pharmacological therapy data elements	0	20	0
Laboratory results elements	10	0	1
Diagnostic and therapeutic procedures elements	2	2	26
Outcomes data elements	0	0	2

Exact matches are available in both lists; no matches means that the data field is not represented in the Data Inventory and ‘data field as value of a data element’ means that data fields can be matched to data elements because they refer to similar concepts (for example, data field ‘Diabetes’ corresponds to data elements ‘Diagnosis/Text’).

In contrast to our work, the ‘key data elements of a base cardiovascular vocabulary’ describe elements that should be documented in EHRs to support the exchange of information throughout care, while the Data Inventory is a catalog of available data elements in EHRs that are important for clinical research.

Häyrinen *et al*. [19] describe the core data elements that were introduced in Finland for a national electronic health record. Similar to our approach Häyrinen *et al*. defined a list of data elements, added a definition to each item and furthermore added the terminology or code systems that should be used, if suitable systems were existent. Häyrinen’s list contains elements that would be implemented for the national EHR. In contrast, for our Data Inventory we identified elements that are currently documented in EHRs. A comparison of the ‘core data elements’ and the data elements of the Data Inventory showed several similarities: for example ‘health problems and diagnosis’ should use ICD-10 or ICPC (*International Classification of Primary Care*) codes which correlates to ‘diagnosis/code’ of the Data Inventory. We did not specify which classification should be used, but ICD-10 or ICPC codes would be values of this data element as well.

Weng *et al*. [12] and Luo *et al*. [13] describe in related publications, a semi-automatic approach that allows annotating free text eligibility criteria using semantic representation. In contrast to our expert-driven simplification approach - intended to reduce complexity with a focus on trial feasibility - those methods aim at semi-automatically extracting the complete information out of free text. Due to the different approaches, there is some overlap with our Data Inventory. Out of the 27 semantic classes from the Weng and Luo publications, only ‘Age’ and ‘Gender’ match directly, ten classes correspond to one or more data groups and 15 are not represented in the Data Inventory at all. Table 5 shows how the semantic classes correspond to the data groups.

**Table 5:** **Semantic classes from Weng**[12]**/Luo**[13]**corresponding to the data groups**

**Data groups (this work)**	**Semantic classes (Weng et al./Luo *et al*.)**
Diagnosis	Disease, Symptom and signs, Neoplasm status
Procedures	Therapy or surgery, Diagnostic or lab results
Laboratory findings	Diagnostic or lab results
Findings	Diagnostic or lab results
Medical history	Pregnancy-related activity, Addictive behavior
Scores and Classification	Neoplasm status, Disease stage
Medication	Pharmaceutical substance or drug
Demographics	Age, Gender

Comparison between data groups of this work and semantic classes according to Weng [12]/Luo [13]. Some of the semantic classes are listed more than once because they correspond to more than one data group. Similarly, one data group can correspond to one or more semantic class.

Köpcke *et al*. [20] describe in their work the data completeness of five German hospital EHRs from data elements of 15 studies re-using Luo’s semantic classes. Köpcke’s and our work show similar tendencies for completeness and usage. For example, age and gender are highly available and used for both lists, although information with respect to pregnancies is not so readily available in both. Although [20] is focused on patient recruitment and the Data Inventory on feasibility, the tendencies of availability and usage are similar.

### Lessons learned

Eligibility criteria in clinical trial protocols are usually described using long and complicated free text sentences which cannot readily be used for further processing. Through a process of simplifying the criteria, the information content can be reduced or split up in several parts until single data elements are left, which can be represented in a formal, consistent way. When doing the simplification, we also identified a difference between required data elements for trial feasibility and recruitment. While the criteria for feasibility are fewer in number and more general, data elements for recruitment have to be more precise. From the experiences made of the simplification task, best practice principles for simplifying eligibility criteria [8] were created. They describe how eligibility criteria should be formulated to be clearly understandable and computer readable with little additional effort. When comparing the Data Inventory with billing data, in particular DRG data [21], one can see that EHRs nowadays already contain more data elements that can be re-used for research than just diagnosis and procedure codes; laboratory findings, for example.

Weiskopf and Weng [22] identified five common dimensions of data quality reviewing 95 articles (completeness, correctness, plausibility, concordance and currency). Evaluating correctness, plausibility and currency is a laborious task which requires medical knowledge and can therefore not be automated readily. To investigate correctness for example, patient charts would have to be checked manually to determine if the documented data are correct. A mapping between the data export and the elements in the EHRs was done with knowledge of the local project partners; the concordance however was not evaluated in detail. However, through the data exports we did capture the availability of the data elements (completeness) as is shown in Figure 2. During the creation of the Data Inventory, we have seen in several instances that data quality is a critical issue. Automatic transfer of EHR data into an electronic data capture system for example, can only be performed if the data quality is ‘high enough’. The aim of the inventory, however, was not to address these issues but to obtain an overview of what is available and what is required.

### Limitations

Certain disease areas (for example, diabetes or inflammatory diseases) are not yet fully covered by the Data Inventory (see Table 2). The current version is focused on site feasibility, so data elements for patient recruitment or clinical trial execution are not considered. Our approach was to create a global Data Inventory based on relevant elements for research that are available in EHRs. It does not take into account varying documentation needs of different disease areas, but should be more easily implemented in contrast to several disease-specific data inventories.

### Outlook

We expect the Data Inventory to be constantly evolving based upon future releases of EHR systems and analyses of more trials from different disease areas. In a review of 89 papers, Häyrinen [23] reported that use of information systems leads to more complete and detailed documentation, and structured data entry increases completeness and accuracy of data. Future work will focus on appropriate methods and procedures to further improve EHR data completeness and include those data elements in routine care that are currently missing. Scope, as well as benefits and costs need to be taken into account when aiming to include new elements into the routine documentation. The data quality of EHRs in general, not only the completeness, will need to be analyzed to ensure that the data can be used for clinical studies.

## Conclusion

Today, EHR systems already provide many data elements that can be used for feasibility analysis of clinical studies. An inventory of elements was created in a combined effort between experts from pharmaceutical companies and academic sites. It provides a common set of data elements that are frequently used in clinical research and at the same time available for re-use from current hospital information systems.

## Abbreviations

DRG: Diagnosis related group; ECOG: Eastern Cooperative Oncology Group; EFPIA: European Federation of Pharmaceutical Industries and Associations; EHR4CR: Electronic Health Records for Clinical Research; EHR: Electronic health record; ICD10: International Statistical Classification of Diseases and Related Health Problems 10th Revision; IIT: Investigator initiated trial; IMI: Innovative Medicine Initiative; LOINC: Logical Observation Identifiers Names and Codes; MMSE: Mini-Mental State Examination; NCIm: NCI Metathesaurus; UMLS: Unified Medical Language System.

## Competing interests

The authors declare that they have no competing interests.

## Authors’ contributions

JD helped with the simplification, was part of the peer review group, collected the data, analyzed it and wrote the manuscript. MD was part of the peer review group and helped to draft the manuscript. FF helped with the simplification, was part of the peer review group, supervised the methodological approach and helped to draft the manuscript. All authors read and approved the final manuscript.

## Supplementary Material

Additional file 1 **Complete Data Inventory with all data elements.** The Data Inventory contains data elements for feasibility analysis that were extracted from clinical trial protocols and that were verified to be available in European EHR systems. The inventory contains data element concepts (data group + data item), optional examples, the definitions and links to NCIm. Click here for file
